# Observation of room temperature excitons in an atomically thin topological insulator

**DOI:** 10.1038/s41467-022-33822-8

**Published:** 2022-10-23

**Authors:** Marcin Syperek, Raul Stühler, Armando Consiglio, Paweł Holewa, Paweł Wyborski, Łukasz Dusanowski, Felix Reis, Sven Höfling, Ronny Thomale, Werner Hanke, Ralph Claessen, Domenico Di Sante, Christian Schneider

**Affiliations:** 1 Department of Experimental Physics, Faculty of Fundamental Problems of Technology, Wrocław University of Science and Technology, Wybrzeże Wyspiańskiego 27, 50-370 Wrocław, Poland; 2 Physikalisches Institut and Würzburg-Dresden Cluster of Excellence ct.qmat, Universität Würzburg, 97074 Würzburg, Germany; 3 Institut für Theoretische Physik und Astrophysik and Würzburg-Dresden Cluster of Excellence ct.qmat, Universität Würzburg, 97074 Würzburg, Germany; 4 Department of Physics and Astronomy, University of Bologna, 40127 Bologna, Italy; 5 Center for Computational Quantum Physics, Flatiron Institute, New York, NY 10010 USA; 6 Institute of Physics, University of Oldenburg, 26129 Oldenburg, Germany

**Keywords:** Topological insulators, Two-dimensional materials

## Abstract

Optical spectroscopy of ultimately thin materials has significantly enhanced our understanding of collective excitations in low-dimensional semiconductors. This is particularly reflected by the rich physics of excitons in atomically thin crystals which uniquely arises from the interplay of strong Coulomb correlation, spin-orbit coupling (SOC), and lattice geometry. Here we extend the field by reporting the observation of room temperature excitons in a material of non-trivial global topology. We study the fundamental optical excitation spectrum of a single layer of bismuth atoms epitaxially grown on a SiC substrate (hereafter bismuthene or Bi/SiC) which has been established as a large-gap, two-dimensional (2D) quantum spin Hall (QSH) insulator. Strongly developed optical resonances are observed to emerge around the direct gap at the K and K’ points of the Brillouin zone, indicating the formation of bound excitons with considerable oscillator strength. These experimental findings are corroborated, concerning both the character of the excitonic resonances as well as their energy scale, by ab-initio *GW* and Bethe-Salpeter equation calculations, confirming strong Coulomb interaction effects in these optical excitations. Our observations provide evidence of excitons in a 2D QSH insulator at room temperature, with excitonic and topological physics deriving from the very same electronic structure.

## Abstract (web summary)

Here, the authors report the observation of room temperature excitons in a single layer of bismuth atoms epitaxially grown on a SiC substrate - a material of non-trivial global topology - with excitonic and topological physics deriving from the very same electronic structure.

## Introduction

Optical spectroscopy conducted on atomically thin sheets of transition metal dichalcogenides (TMDCs) has opened a broad spectrum of fundamental research, and significantly enhanced our understanding of the physics of elementary excitations in low-dimensional semiconductors^1,2^. Indeed, the dramatic increase of Coulomb correlations in two-dimensional structures has revealed the emergence of a vast variety of many-body states, including non-hydrogenic Rydberg series of excitons^3^, stable charged-^4,5^, and multi-excitonic complexes^6,7^.

Our observation of excitons in a large-gap QSH insulator at room temperature opens up a new exciting avenue of combining excitonic physics and topological electronic properties. Here a first central step was recognizing that the optical selection rules are governed by the winding number of the low-energy gapped Dirac bandstructure, which like the Berry curvature, is a "local" topological quantity in *k*-space^8–11^. This local physics is indeed determined by the Berry curvature flux through the small *k*-space spanned by the relative motion of the *e*–*h* pair in the excitonic wavefunction^12^. This appears, e.g., in a class of materials called gapped chiral fermion systems, which comprises gapped topological surface states^13^, monolayers of TMDCs^14^ and biased bilayers with broken inversion symmetry^15^. Recent work indeed has connected the strength and required light polarization of an excitonic transition with the corresponding optical matrix elements’ winding number^16–18^, and thus successfully described the canonical valley-contrasting selection rules of K and K’ excitons in TMDCs.

However, despite the fact that optical properties of TMDCs thus are strongly impacted by the above mentioned local *k*-space phenomena ^8^, the commonly investigated pristine monolayer TMDCs WS_2_, WSe_2_, MoS_2_, MoSe_2_, and MoTe_2_, in their 2H-phase, belong to the class of topologically trivial insulators. From a fundamental perspective, the implications of the interlink between optical selection rules and topological band invariants (a direct link between excitonic physics and global topology, i.e., the Chern number as *k*-space integral over the Berry curvature) are expected to trigger a plethora of future experimental and theoretical investigations. First steps in this direction are discussed in our conclusions and in the Supplementary Information.

To date, though, any experimental scrutiny of the impact of non-trivial global band topology on light-matter interaction and exciton physics has so far been hampered by the lack of suitable topological insulators with sufficiently wide band gap to allow coupling to high-energy photons. It is only very recently that such 2D topological insulators have been discovered and synthesized, namely 1T’-WTe_2_ monolayers^19^ and Bi/SiC, an atomically thin bismuth layer, which is arranged in a graphene-like unbuckled honeycomb lattice^20^. In bismuthene, the SOC induced by the heavy Bi atoms and the orbital filtering resulting from the covalent bonding to the substrate are responsible for the emergence of a giant topological band-gap^21^, which is large enough to be probed by optical spectroscopy in the near infrared. This is in striking contrast to any other topological insulator previously known, and opens up novel experimental opportunities to the study of photo-induced excitonics in 2D topological insulators.

Here, we study the electronic as well as the optical band-gap of Bi/SiC employing photo-modulated reflectivity and scanning tunneling spectroscopy (STS) combined with ab-initio *GW* calculations, accounting for the electronic Coulomb interactions^22–24^. Our investigation reveals the emergence of strong optical resonances within the electronic band-gap which we associate with the material’s excitonic modes. The experimentally derived excitonic resonance energies are notably well reproduced by many-body calculations in the *GW*+Bethe–Salpeter equation framework^25–29^. Our findings, which evidence the emergence of excitons at room temperature in a large-gap QSH insulator, ideally advance present activities to link excitonics and topological materials research ^16–18,30–38^.

## Results I: electronic band structure and single-particle band gap

**Fig. 1:**
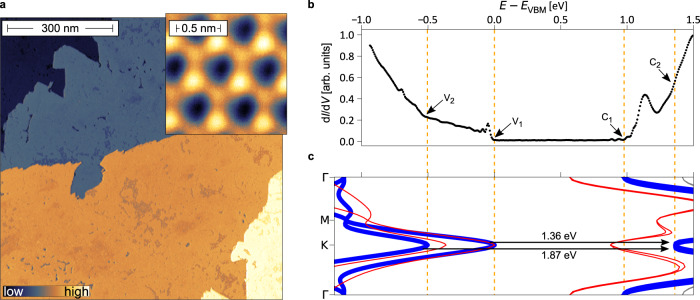
Single-particle electronic properties of Bi/SiC. **a** Constant current STM image of bismuthene on SiC(0001). The topographic map displays the high coverage (~87%) of the epitaxially grown Bi monolayer and the terrace structure of the underlying SiC substrate. (*T* = 4.2 K and *V*_set_ = 2.6 V, *I*_set_ = 50 pA.) Inset: A high-resolution measurement reveals the bismuthene honeycomb lattice. (*T* = 4.2 K and *V*_set_ = − 0.4 V, *I*_set_ = 200 pA.) **b** Differential tunneling conductivity (d*I*/d*V*) spectrum, locally measured in the center of the bismuthene film (*T* = 4.2 K and *V*_set_ = − 0.4 V, *I*_set_ = 200 pA, ${V}_{{{{{{{{\rm{mod}}}}}}}}}=10\,{{{{{{{\rm{mV}}}}}}}}$). Note that the peak at ≈ 1.1 eV is not an intrinsic feature of the bismuthene film, but originates from a local defect state in the SiC substrate (see Section II of the Supplementary Information). **c** *GW* band structure calculation (blue curves) for bismuthene. The direct optical transitions at the K-point are indicated by arrows. Also shown is the DFT band structure obtained with the HSE hybrid functional (red curves)^20^, illustrating the propensity of DFT to underestimate the band gaps. Characteristic points in the spectrum that correspond to the valence band onsets (*V*_1_, *V*_2_) and the conduction band onsets (*C*_1_, *C*_2_) are marked.

The bismuthene monolayers used in our optical experiments were grown by molecular beam epitaxy using a Knudsen cell as Bi source and SiC(0001) as supporting substrate (for details see ref. 20 and Methods). The high quality of the resulting layers is best visualized by scanning tunneling microscopy (STM). The topographic overview scan in Fig. 1a, obtained by constant-current STM, shows an excellent Bi coverage of ~87%. The inset depicts a detailed view on the honeycomb arrangement of the Bi atoms, ultimately confirming successful film growth.

For a detailed discussion of the optical excitations and related exciton physics it is mandatory to first develop a good understanding of the underlying single-particle electronic properties of Bi/SiC. Detailed mapping of the valence bands has already been performed by angle-resolved photoelectron spectroscopy (ARPES) and found to be in convincing agreement with density-functional theory (DFT)^20^. However, here we are interested in both occupied and unoccupied bands and their relative energy separation which is relevant for the optical transitions. This can be probed by STS which records the differential tunneling conductivity as a measure of the electronic local density of states (LDOS).

Figure 1b displays the STS spectrum measured at the center of the bismuthene sample where it shows its highest structural quality. The most prominent feature of the spectrum is the vanishing LDOS between the energy positions marked by *V*_1_ and *C*_1_. This region is identified as the fundamental band gap, with the spectral onsets *V*_1_ and *C*_1_ representing the valence band maximum (VBM) and conduction band minimum (CBM), respectively. For a quantitative analysis the onset energy *V*_1_ is evaluated by extrapolating the valence band edge to the zero conductance plateau (see Supplementary Information), an established method to determine gap edges in STS. The same procedure is applied to the CBM at *C*_1_. From the energy separation of *C*_1_ and *V*_1_ we infer an experimental single-particle band-gap *E*_ind_ of 0.80 eV ≤ *E*_ind_ ≤ 0.96 eV.

In theory, the established method for a reliable estimate of the single-particle gap is the *GW* approximation, which expresses the electronic self-energy Σ by the electron propagator *G* and the screened Coulomb interaction *W*(**k**, *ω*) via Σ = *iGW*^22,24,39^. This goes beyond DFT-type approaches, which are designed to yield accurate total (ground-state) energies but are not devised for extracting single-particle energies^40^ and often tend to underestimate band gaps (see Fig. 1c, red curves for an example).

The *GW* band structure for Bi/SiC (see Methods for the details) is shown in Fig. 1c as solid blue lines. It is characterized by strongly Rashba-split bands at the K (K’) valleys and an indirect electronic band gap between the VBM at K and the CBM at Γ of *E*_ind_ = 0.97 eV, in excellent agreement with our experimental gap value. The *GW* band structure also accounts for some other features in our experimental STS data, as indicated by the dashed orange lines connecting Fig. 1b, c. For instance, the kink in the measured LDOS at approximately −0.5 eV (marked by *V*_2_) matches almost perfectly with the maximum of the lower Rashba-split *GW* valence band. Likewise, the experimental kink- feature *C*_2_ agrees well with the K-point minimum of the *GW* conduction band. Finally, and as of immediate relevance for the optical transitions, the direct single-particle gap at the K (K’) valley amounts to *E*_direct_ = 1.36 eV in our *GW* calculation.

## Results II: optical spectroscopy, optical gap and excitonic resonances

Next, we expand our study to the optical response, i.e., to the two-particle excitations. Experimentally, bismuthene’s fundamental optical excitation spectrum is probed via highly spatially-resolved photo-modulated reflectivity (PR). PR is a very sensitive probe that has been successfully applied to study the excitonic spectrum of atomically thin crystals^41,42^, since it directly probes the derivative of the absorption spectrum with respect to energy, in stark contrast to direct reflectivity techniques.

**Fig. 2:**
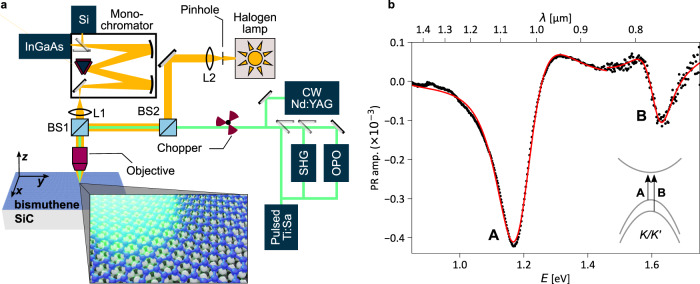
Optical spectroscopy of Bi/SiC. **a** The experimental setup for high-spatial-resolution photo-modulated reflectivity (PR). **b** The fundamental excitation spectrum of bismuthene measured in the PR experiment at *T* = 300 K (CW excitation, *E*_laser_ ~ 2.33 eV, *P*_ave_ ~ 0.5 mW). *A* and *B* are exciton-like PR features centered at *E*_A_ ≈ 1.19 eV and *E*_B_ ≈ 1.63 eV, respectively. The solid red curve represents a numerical fit with the expression from Eq. (1). Inset: a sketch of the fundamental exciton-like optical transitions at the K/K' points in bismuthene.

The experimental setup is sketched in Fig. 2a (see also Methods). All measurements were performed at room-temperature and with the sample kept under an inert gas atmosphere to prevent surface oxidation of the Bi monolayers. ARPES measurements performed before and after exposure to inert gas confirm that this sample storage method keeps bismuthene chemically intact over weeks (see also Supplementary Information), also owing to the van der Waals-nature of the bismuthene surface. The resulting optical excitation spectrum is displayed in Fig. 2b (see also Supplementary Information). It is dominated by two absorption-like PR spectral features *A* and *B*, located at ~1.19 eV and ~1.63 eV, respectively. In analogy to other two-dimensional semiconducting materials^43^ and, in particular, taking into account the aforementioned agreement between theory and experiment concerning the single-particle properties, it is most reasonable to assign the captured optical transitions to Coulomb-interacting electron-hole excitations in the vicinity of K and K’ points of the Brillouin zone (see inset in Fig. 2b).

For a more quantitative data analysis, we employ an expression for the PR response of excitons based on an adapted model suggested by Shanabrook et al.^44^ (see Supplementary Information). Within this approach, the PR feature acquires the form of the first derivative of the dielectric function:

1 $$\frac{{{\Delta }}R}{R}={\mathfrak{R}}\left({C}_{{{{{{{{\rm{j}}}}}}}}}{e}^{i\theta }{(E-{E}_{{{{{{{{\rm{j}}}}}}}}}+i{{{\Gamma }}}_{{{{{{{{\rm{j}}}}}}}}})}^{-2}\right)\,.$$

Here, *C*_j_ and *θ* are the resonance amplitude and phase, *E*_j_ and Γ_j_ are the energy and broadening parameters of the optical transition, respectively. The fitting procedure to the PR spectrum in Fig. 2b yields *E*_A_ = (1.191 ± 0.004) eV and *E*_B_ = (1.625 ± 0.004) eV, Γ_A_ = (100 ± 4) meV and Γ_B_ = (60 ± 4) meV. It is worth noting that both transitions show substantial spectral broadening, which we attribute to effects arising from, e.g., sample inhomogeneity within the probed area and interaction between excitons and phonons in these room-temperature measurements^45–47^.

**Fig. 3:**
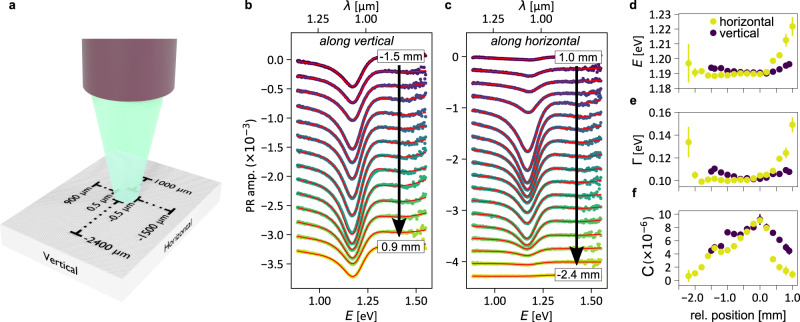
Spatial modulation of the optical response of Bi/SiC. **a** Sketch of the scanning movement along a vertical and horizontal line on Bi/SiC. **b**, **c** Evolution of the PR resonance of the *A*-peak along the vertical and horizontal scanning line, respectively. Solid red lines represent the fitting curves (Eq. (1)). **d**–**f** The energy of the *A* exciton transition, broadening, and the PR amplitude, respectively. (Pulsed laser source, *E*_laser_ ~ 1.65 eV, average pump power: ~0.34 mW, pump fluence: ~0.57 mJ/cm^2^). Error bars are the numerical fit uncertainties. The PR amplitude in horizontal direction correlates well with the bismuthene sample quality (see Supplementary Information).

The high spatial resolution of the PR experiment allows us to probe our sample from the coherent crystal regime in the center of the Bi monolayer to a more disordered regime at its edges. As schematically depicted in Fig. 3a, we follow the signature of the *A*-resonance as the sample is scanned along the indicated vertical and horizontal lines. The corresponding evolution of the PR spectrum is presented in Fig. 3b, c. The fitting procedure reveals that the transition energy (Fig. 3d), broadening (Fig. 3e), and the PR amplitude (Fig. 3f) indeed vary only weakly along the 2 mm-long scanning line, suggesting the fairly good quality of our bismuthene layer over a macroscopically large spatial extension. The layer quality deteriorates close to the bismuthene edges, which is clearly reflected by the diminishing PR amplitude as well as the increased spectral broadening of the resonance. This observation is in line with local structure characterization of our bismuthene films by STM and low-energy electron diffraction (LEED), which reveal reduced crystalline order towards the sample edges (see Supplementary Information). The film inhomogeneity from center to edge is most likely related to a temperature gradient during epitaxial growth. Independent of the detailed mechanism, the marked correlation between PR signal and local crystalline quality is a clear indication that the observed PR resonances are indeed intrinsic to the pristine bismuthene layer.

Turning now to the theoretical picture of the optical response, first we wish to point out that the many-body effects embedded in the single-particle band gap versus those contained in the optical excitation spectrum are of different nature. Even though electronic interactions are already relevant for the self-energy of the single-particle excitations, as, e.g., captured by our *GW* approximation above, the optical spectrum is additionally affected by the Coulomb coupling between electrons and holes generated by the photo-excitation, lying at the very heart of exciton physics. For a theoretical description of the two-particle properties and the excitation spectrum, including electron-hole interactions, we solve the Bethe–Salpeter equation (BSE) for the electron-hole amplitude^25,26,28^:

2 $$({E}_{c{{{{{{{\bf{k}}}}}}}}}-{E}_{v{{{{{{{\bf{k}}}}}}}}}){A}_{vc{{{{{{{\bf{k}}}}}}}}}+{{{\Sigma }}}_{v^{\prime} c^{\prime} {{{{{{{\bf{k}}}}}}}}^{\prime} }\langle vc{{{{{{{\bf{k}}}}}}}}|{K}_{eh}|v^{\prime} c^{\prime} {{{{{{{\bf{k}}}}}}}}^{\prime} \rangle {A}_{v^{\prime} c^{\prime} {{{{{{{\bf{k}}}}}}}}^{\prime} }={{\Omega }}{A}_{vc{{{{{{{\bf{k}}}}}}}}}\,,$$

where the electronic excitations are given in the basis of electron-hole pairs with quasi-particle energies *E*_*v***k**_ and *E*_*c***k**_ in the valence and conduction bands. The *A*_*vc***k**_ are the coefficients of the excitons in the electron-hole basis and Ω are the eigen-energies. The kernel *K*_*eh*_ accounts for the screened Coulomb interaction between electrons and holes, and the exchange interaction, including also local field effects, which are due to the optical excitations of the periodically arranged atomic orbitals in Bi/SiC^48^.

**Fig. 4:**
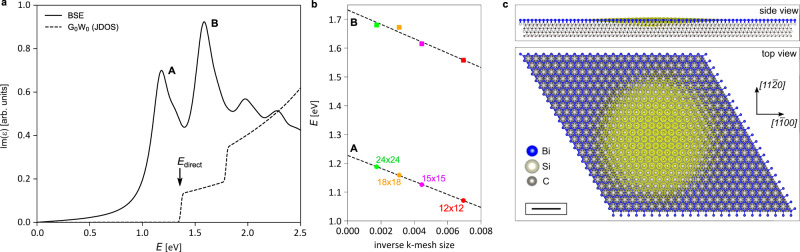
Ab-initio *GW*+BSE results. **a** The solid line displays the absorption spectrum of Bi/SiC inferred from the imaginary part of the dielectric function from the BSE calculation. The dashed line displays the joint density of states from the *GW* calculation, where the electron-hole interaction is neglected. **b** Convergence of the *A* and *B* exciton peaks as a function of the inverse of the number of *k*-points. The intersection of the gray dashed line with the *y*-axis gives the result ideally obtained from an infinitely dense *k*-mesh. **c** Lateral and top views of the *A* exciton wave-function. The radius of the exciton is ~3 nm.

The absorption spectra calculated with and without electron-hole interactions are displayed in Fig. 4a. From these calculations, we can extract an excitonic binding energy of ~0.15 eV for the lowest excitonic peak *A*, estimated as the energy difference between the independent particle *GW* absorption gap *E*_direct_ indicated by the arrow and the energy position of peak *A*. The second evident peak *B* is shifted by an energy comparable to the Rashba splitting of the valence states at the K and K’ valleys. This results in a significant separation between the *A* and *B* excitonic peaks, and the eventual merging of the latter into the continuum of electron-hole excitations (forming a Fano resonance depending on coupling strength between exciton *B* and continuum).

We find that the position of the theoretical *A* and *B* peaks is highly sensitive to the number of *k*-points employed in the BSE calculation. Figure 4b shows the convergence for several *k*-meshes, highlighting a linear scaling towards *E*_A_ ≈ 1.23 eV for an infinitely dense sampling, in excellent agreement with its measured value (1.19 eV). For exciton peak *B*, the extrapolation yields a theoretical value of *E*_B_ ≈ 1.73 eV, still satisfyingly close to the experimental result (1.63 eV).

The aforementioned convergence issue arises from the extended nature of the Wannier-Mott type excitons in Bi/SiC. In Fig. 4c, we give side and top views of the *A* peak excitonic wave-function obtained from our calculations. The exciton radius of ~3 nm extends over several lattice constants, resulting from the strong localization in reciprocal space around the K and K’ valleys. This, in turn, requires a dense *k*-mesh to reduce the spurious Coulomb repulsion between periodic excitons. Remarkably, Fig. 4c shows also how the exciton wave-function is not strictly localized inside the Bi monolayer but rather extends for at least two atomic layers into the SiC substrate. This further evidences the pivotal role played by the substrate in bismuthene at the single- and two-particle levels: SiC is not only responsible for the orbital filtering mechanism and the huge topological gap^20,21^, but also provides an important screening channel for the Coulomb interaction.

## Discussion

In conclusion, we report the observation of excitons in a large-gap, atomically thin QSH insulator at room temperature. We capture two prominent transitions in the many-particle response of bismuthene, which we quantitatively associate with excitonic transitions from the Rashba-split valence bands. It is important to emphasize that the many-particle response of bismuthene differs fundamentally from what is known from the vastly studied 2H-TMDCs. First, whereas in the latter materials the low-energy interband transitions are captured by two decoupled gapped chiral Dirac fermion models^16,17^ with the same winding number in each valley (with opposite sign between K/K’-valley), in bismuthene, low-energy interband transitions are described by chiral models with different winding numbers in each valley. Therefore, in contrast to the strict valley-locked optical selection rules that govern 2H-TMDCs, bismuthene is sensitive to controlling the coupling to *σ*^+^- and *σ*^−^-polarized light by reordering electronic bands with external electric and magnetic fields^18^ (see also Supplementary Information). The latter provides a clear technological advantage over TMDCs for engineering electro-optical devices based on atomically thin materials. Second, in bismuthene the excitonic *and* the topological physics originate from the very same electronic states, i.e., they establish a direct link between excitonic physics and topology, which has only been proposed theoretically so far (see for example, ref. 49). We foresee that our findings trigger a plethora of future experimental investigations, for example related to the helical excitonic transport in topological edge modes^35^, finding and assessing "global" topological properties of the QSH phase via optical band-to-band transitions and selection rules (see also Supplementary Information), topological polaritonics^50^, and other exotic many-body quantum phases.

## Methods

### Sample preparation

The bismuthene sample was grown by molecular beam epitaxy using a Knudsen cell as Bi source and SiC(0001) as a supporting substrate. During growth, the temperature of the SiC substrates was controlled via direct current resistive heating. The characteristic of this heating method is a temperature gradient of ~20 °C along the longitudinal extent of the sample (the horizontal direction). As bismuthene growth is sensitive to such a narrow temperature window, we observe a strong correlation of bismuthene film coverage with the spatial position in the horizontal direction. Based on low energy electron diffraction (LEED), the bismuthene film quality can be macroscopically checked. High intensity of the $\sqrt{3}\times \sqrt{3}$ spots associated with the bismuthene reconstruction on SiC(0001) and low diffuse background intensity provide a qualitative measure of film quality (see Supplementary Information).

### Optical setup

In the PR experiment (Figs. 2a and 3a, c), the sample is kept under a N_2_ inert gas atmosphere (<0.1 ppm H_2_O;<0.1 ppm O_2_) and mounted on an x-y-z mechanical stage, allowing for selecting the probed sample’s area with a ~100 nm accuracy in each spatial direction. The infinity-corrected microscope objective (0.42 numerical aperture and 20 mm working distance) used in the PR experiment operates in the spectral range of 0.4–1.8 μm with ×20 magnification. The focal plane of the modulation laser beam is inspected through the imaging setup with a CCD camera showing the sample surface, the white light spot, and the laser spot. Since the modulation laser spot is roughly 1 μm in diameter, it defines the total area of a sample tested in a single experimental run. White light is provided by a Halogen lamp. A mechanical chopper modulates the laser beam with a 50%:50% duty cycle at an *f*_m_ frequency depending on the utilized photon detector: (i) the *f*_m_ = 10 Hz for the Si diode, sensitive in the 0.3–1.0 μm spectral range, or (ii) *f*_m_ = 270 Hz for the InGaAs photo-diode, covering the spectral range of 0.9–1.5 μm. Several types of laser sources are employed: (a) a continuous-wave (CW) neodymium-doped yttrium aluminum garnet laser emitting at 532 nm photon wavelength; (b) a Ti: Sapphire oscillator, providing a train of ~140 fs-long-pulses at 76 MHz repetition frequency, spectrally tunable in the range of 0.7–0.95 μm or 0.35–0.475 μm (second harmonic generation), and (c) a synchronously-pumped optical parametric oscillator providing a train of ~200 fs-long-pulses spectrally tunable in the range of 0.5–0.76 μm or 0.97–1.1 μm. Spectral light analysis is provided by a spectrometer consisting of a 0.3 m-focal-length grating monochromator (0.2 nm ultimate spectral resolution) and two detectors mentioned above. The spectral resolution is limited to an arbitrarily chosen monochromator scanning step in the PR experiment, which is ~4 meV. The lock-in amplifier reads the detector signal at the *f*_m_ carrier frequency.

### Theoretical framework

The approach to determining and quantifying the importance of the electron-hole interaction, and corresponding excitonic effects, in Bi/SiC employed the following methods (in this hierarchical order): *DFT* → *GW* → *BSE*. Density Functional Theory (DFT). It is used to understand the ground-state properties, with the interacting many-electrons problem being mapped onto a non-interacting electrons problem. Spectroscopic properties, however, involve excited particles above the ground state and a correct step in this direction can be done by considering the many-body electron-electron interaction in the framework of *GW* approximation. Starting from DFT single-particle energies, one can compute the Quasi-Particle (QP) energies by taking into account the self-energy Σ expressed through the single-particle Green’s function *G* and the screened Coulomb interaction *W* (here computed within the random phase approximation RPA including local field effects). This approximation is known to be correct at about 0.1 eV^51^, which makes our *GW* results overall consistent with all our STS spectra shown in the manuscript. Finally, on top of the *GW* results, the effect of the electron-hole interactions is included using the Bethe–Salpeter Equation (BSE) and the excitonic two-body wave-function is written via a product of occupied and unoccupied orbitals. The results here presented have been obtained using the simulation packages Quantum Espresso^52^ and YAMBO^53,54^. Norm-conserving pseudo-potentials are used to quantify the electron-ion interactions and the Perdew–Burke–Ernzerhof (PBE) functional^55^ is used for the exchange-correlation potential. Wave-functions are expanded in plane-waves with an energy cut-off of 40 Ry, large enough to guarantee converged results. The Brillouin zone is then sampled with a 12 × 12 × 1 grid for DFT ionic relaxation and non self-consistent calculations, but it has been increased up to 24 × 24 × 1 *k*-points for many-body calculations. Spin-orbit coupling is included self-consistently. QP energies are then computed using a single shot *G*_0_*W*_0_ approximation.

## Supplementary information

Supplemantary Informations

## Data Availability

The data that support the findings of this study are available from the corresponding authors upon reasonable request.

## Code availability

The codes of this study are available from the corresponding authors upon reasonable request.
