# Supplementary material for: Observation of room temperature excitons in an atomically thin topological insulator
Source: Nat Commun. 2022 Oct 23;13:6313. doi: 10.1038/s41467-022-33822-8 (PMC9588767; doi:10.1038/s41467-022-33822-8)
Supplement: Supplementary file 1 — Supplemantary Informations [file 41467_2022_33822_MOESM1_ESM.pdf]

Supplementary Information

—

Observation of room temperature excitons in an atomically thin topological insulator

Marcin Syperek,[1,*] Raul Stühler,[2,*] Armando Consiglio,[3,*] Paweł Holewa,[1] Paweł Wyborski,[1] Łukasz Dusanowski,[1,2] Felix Reis,[2] Sven Höfling,[2] Ronny Thomale,[3] Werner Hanke,[3] Ralph Claessen,[2,†] Domenico Di Sante,[4,5,‡] and Christian Schneider[6,§]

[1]*Department of Experimental Physics, Faculty of Fundamental Problems of Technology, Wrocław University of Science and Technology, Wybrzeże Wyspiańskiego 27, 50-370 Wrocław, Poland*
[2]*Physikalisches Institut and Würzburg-Dresden Cluster of Excellence ct.qmat, Universität Würzburg, 97074 Würzburg, Germany*
[3]*Institut für Theoretische Physik und Astrophysik and Würzburg-Dresden Cluster of Excellence ct.qmat, Universität Würzburg, 97074 Würzburg, Germany*
[4]*Department of Physics and Astronomy, University of Bologna, 40127 Bologna, Italy*
[5]*Center for Computational Quantum Physics, Flatiron Institute, New York, NY 10010, USA*
[6]*Institute of Physics, University of Oldenburg, 26129 Oldenburg, Germany*

(Dated: October 4, 2022)

CONTENTS

Supplementary Sec. I. Spatial variation of bismuthene film quality 1

Supplementary Sec. II. Substrate-related contribution to the STS spectra: the role of localized SiC defect states 3

Supplementary Sec. III. Sensitivity of the bismuthene films to and protection from oxidation 4
A. Oxidation effects in the photoreflectivity experiments 4
B. XPS characterization of bismuthene oxidation and proof of efficient inert gas protection 4
C. Confirmation of efficient inert gas protection by valence band ARPES 7

Supplementary Sec. IV. Derivation of the photoreflectivity lineshape 8

Supplementary Sec. V. Power dependence of the photoreflectivity measurements 9

Supplementary Sec. VI. Determination of single-particle band onsets from STS 9

Supplementary Sec. VII. Topological nature of excitons in Bi/SiC 10

References 11

* These authors contributed equally: Marcin Syperek, Raul Stühler and Armando Consiglio.
† claessen@physik.uni-wuerzburg.de
‡ domenico.disante@unibo.it
§ christian.schneider@uni-oldenburg.de

Supplementary Sec. I. SPATIAL VARIATION OF BISMUTHENE FILM QUALITY

For the preparation of the bismuthene film we use DC resistive heating provided by a standard *ScientaOmicron*-type sample holder (see Supplementary Fig. S1a). Owing to this heating technique, a slight temperature gradient along the horizontal direction of the sample is present during growth and can be monitored by an optical pyrometer. Because the bismuthene epitaxy is sensitive to small temperature variations ($\sim 10\,^{\circ}$C), we observe spatial variations of the bismuthene film quality along the horizontal direction on a mm length scale. The bismuthene film quality can be macroscopically probed by low energy electron diffraction (LEED). In Supplementary Fig. S1b we show a series of LEED images measured at several spots along the horizontal direction of the sample. This allows us to directly map the spatial position dependence of the crystalline sample quality. Clearly, at the center of the sample ($\sim 0.0\,$mm) the bismuthene film quality reaches its maximum. Here we observe the highest intensity of the $\sqrt{3} \times \sqrt{3}$ spots (marked by a blue shaded rhomb) associated with the bismuthene reconstruction on SiC(0001) and the lowest diffuse background intensity.

Towards the left edge of the sample we detect an additional $(2\sqrt{3} \times 2\sqrt{3}R30^{\circ})$ surface reconstruction with weak indications of stripes (marked by a green shaded rhomb and green dashed lines). We attribute this surface phase to a local growth temperature exceeding its optimum value. STM constant current images taken at such spots (see Supplementary Fig. S1c) reveal a reduction of the bismuthene coverage compared to optimal growth temperature (see Fig. 1a). Closer inspection of an area where bismuthene and the high-T reconstruction coexist (see Supplementary Fig. S1d) indeed suggest that the high-T reconstruction is composed of a sub-monolayer. The correspondence of the high-T reconstruction observed in STM with LEED is confirmed by the Fourier transform (FFT) of the real-space STM image, see inset of Supplementary Fig. S1d. Importantly, the spatial variation of the PR amplitude documented in the paper (see Fig. 3c, which probes the same horizontal line as Supplementary Fig. S1b) directly correlates with the bismuthene quality inferred from the LEED measurements.

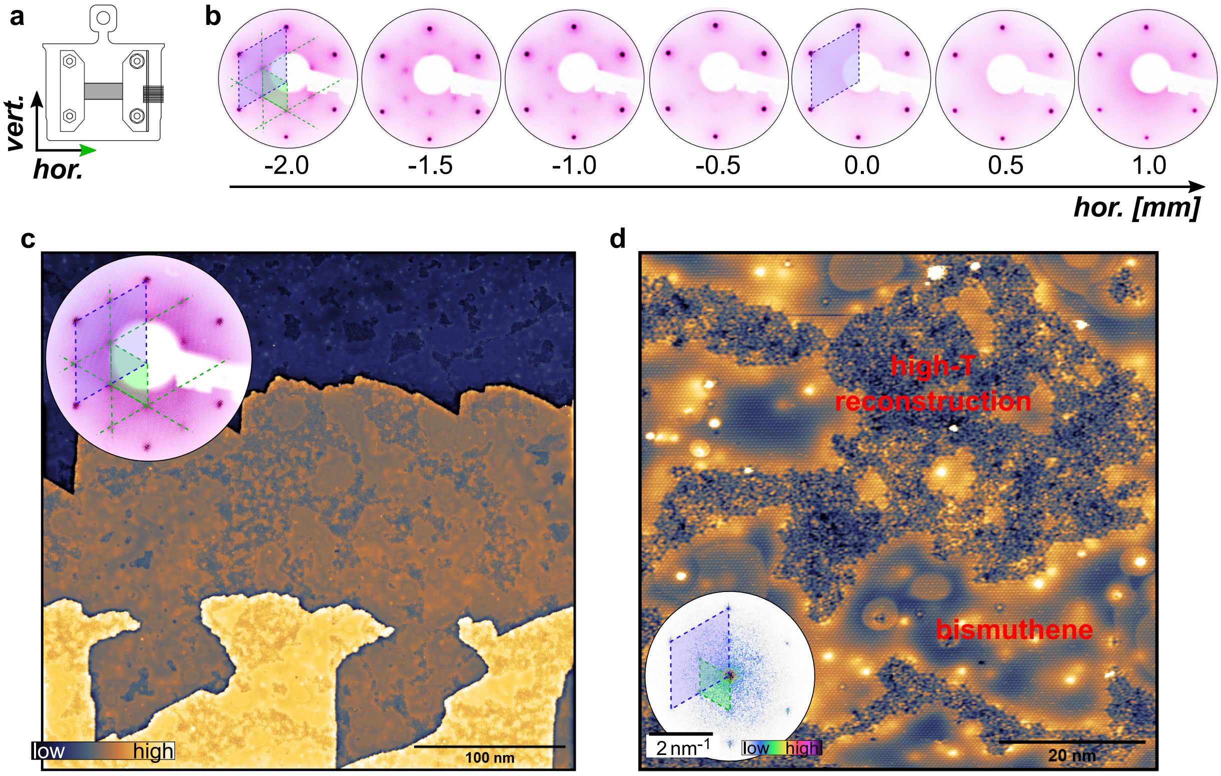

Supplementary Fig. S1. **LEED series along the horizontal direction of the bismuthene sample. a,** Schematic drawing of the bismuthene sample on the SiC substrate (gray strip) mounted on a *ScientaOmicron* DC sample holder. The horizontal direction is defined as the direction along the SiC wafer strip. **b,** LEED series ($E_{\mathrm{kin}} = 40\,\mathrm{eV}$, $T = RT$) along the horizontal direction of the bismuthene sample. The blue-shaded rhombs indicate the surface Brillouin zone (SBZ) defined by the Bragg peaks of the intrinsic bismuthene ($\sqrt{3} \times \sqrt{3}R30°$) reconstruction on the SiC(0001) surface. Towards the left side of the sample additional Bragg peaks are seen to appear, resulting in a new ($2\sqrt{3} \times 2\sqrt{3}R30°$) reconstruction, which is associated with a different (non-bismuthene) phase. The green-shaded rhomb marks the corresponding SBZ. In comparison, the right-hand side of the film displays only intrinsic bismuthene Bragg spots which are however accompanied by a strong diffusive background indicating a highly defective film. The optimal bismuthene coverage is thus located at the center of the film (horizontal position 0.0 mm). **c,** STM constant current image at a spot, where bismuthene and the high-T reconstruction coexist. The bismuthene coverage at this high-T spot is reduced compared to a growth with optimal growth temperature (see Fig. 1a), which can be seen from the large defective areas (these areas appear darker in color). ($T = 4.2\,\mathrm{K}$ and $V_{\mathrm{set}} = 3.0\,\mathrm{V}$, $I_{\mathrm{set}} = 50\,\mathrm{pA}$.) Inset: The LEED image shows additional ($2\sqrt{3} \times 2\sqrt{3}R30°$) Bragg spots with weak indications of stripes (marked by a green shaded rhomb and green dashed lines). ($E_{\mathrm{kin}} = 50\,\mathrm{eV}$, $T = RT$) **d,** High resolution STM constant current image of a surface area like in **c**. The correspondence of the high-T reconstruction observed in STM with the LEED image is revealed by the Fourier transform (FFT) of the real-space STM image, see inset. ($T = 4.2\,\mathrm{K}$ and $V_{\mathrm{set}} = 2.3\,\mathrm{V}$, $I_{\mathrm{set}} = 100\,\mathrm{pA}$.)

Supplementary Sec. II. SUBSTRATE-RELATED CONTRIBUTION TO THE STS SPECTRA: THE ROLE OF LOCALIZED SIC DEFECT STATES

In Supplementary Fig. S2a we show STS dI/dV spectra measured over a wide spatial range spanning multiple unit cells of the pristine bulk bismuthene lattice shown in Supplementary Fig. S2b. Each dI/dV spectrum in Supplementary Fig. S2a represents an average over spectra measured in a 0.5 nm × 0.5 nm area, as marked by the red grid lines in the constant current STM image in Supplementary Fig. S2b. We concentrate on the bias voltage region marked by red dashed lines in Supplementary Fig. S2a which is located near the conduction band onset. It is apparent that there is a particularly strong variation in this bias voltage region of the spectrum. Whereas it shows a smooth and featureless line shape at some areas as in the spectrum labeled as (3, 4), at other areas the spectra show a more peaky line shape, as for example in the spectrum labeled as (1, 4). The enhanced dI/dV connected to the peak in the indicated bias voltage region in the spectrum labeled as (1, 4) is connected to a locally enhanced LDOS and can also be seen in the constant bias voltage dI/dV map in Supplementary Fig. S2c. At the same time the bismuthene monolayer lattice is absolutely homogeneous and fully intact over the entire area that was measured, see Supplementary Fig. S2b. From this homogeneity one would not expect a locally enhanced LDOS. Thus, the enhanced LDOS which occurs disconnected from the film's intact topography implies that its origin is not intrinsic to the bismuthene film itself. We attribute it to a localized defect or donor state in the SiC substrate instead. The most obvious candidate is the nitrogen donor level of the n-doped SiC substrate, which has a binding energy of approx. 70 meV with respect to the conduction band minimum of the substrate. We note that the hydrogen-like wave-function of the donor level has an effective Bohr radius of a few nm, due to the small band mass and large dielectric constant of SiC. Thus, the STM tip can pick up spectral signal from donor atoms that lie several atom layers beneath the SiC surface.

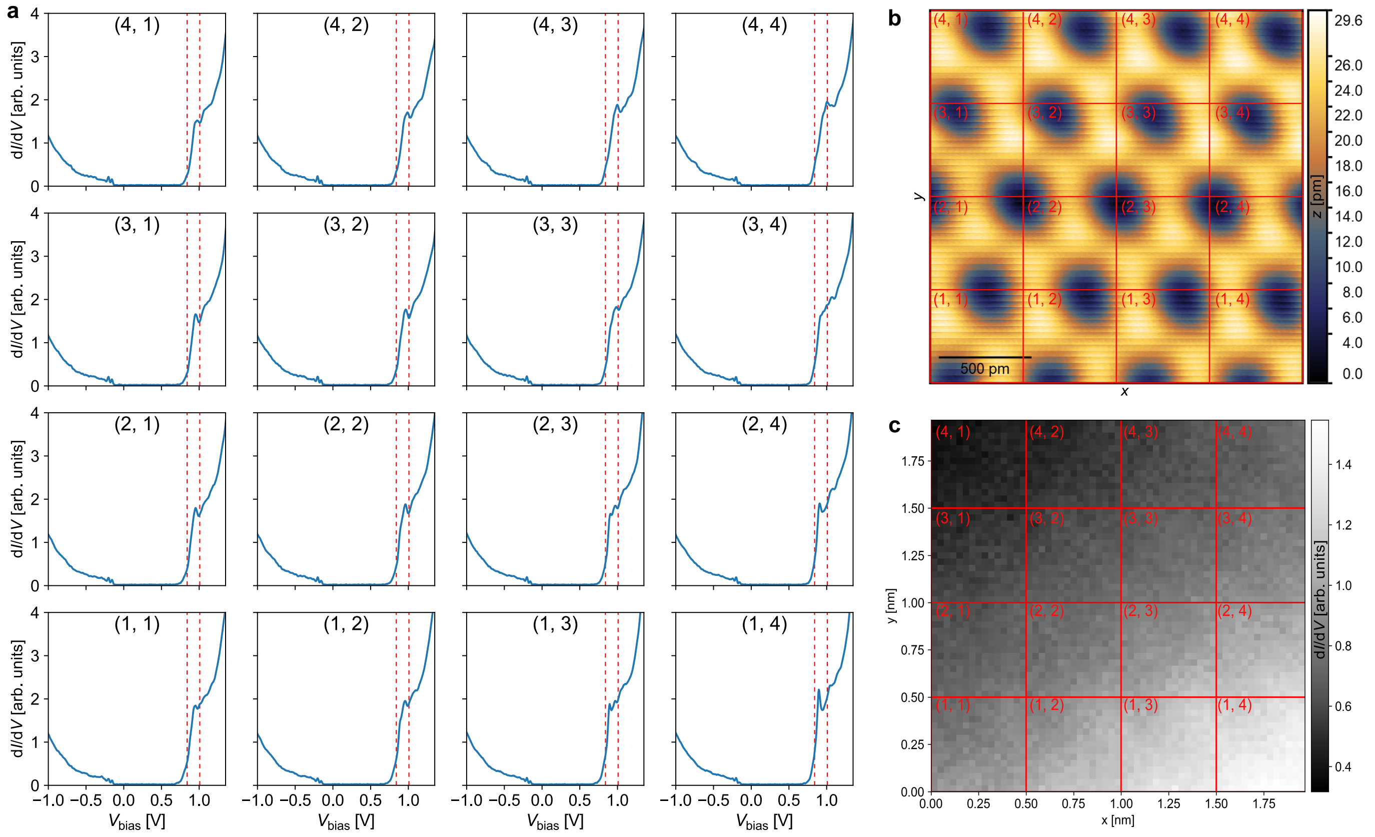

Supplementary Fig. S2. **Local variation of STS spectra.** **a,** Position dependent dI/dV spectra averaged over the respective regions of the grid specified in **b**. The total spatial area reflects the 2 nm × 2 nm bismuthene bulk region depicted in **b** and **c**. **b,** Constant-current map of the measured bulk bismuthene area. The bismuthene lattice is intact throughout the whole area and does not show any topographic irregularities such as point defects. ($T = 4.2$ K and $V_{\mathrm{set}} = -0.5$ V, $I_{\mathrm{set}} = 50$ pA.) **c,** Constant bias voltage dI/dV map averaged over the bias voltage range $V_{\mathrm{bias}} = 0.840 \ldots 1.008$ V as indicated by the red dashed lines in the subpanels of **a**. The higher intensity in the section marked by (1, 4) reflects the peak at the onset of the conduction band states in the corresponding dI/dV spectrum in **a**. ($T = 4.2$ K and $V_{\mathrm{set}} = -1.4$ V, $I_{\mathrm{set}} = 500$ pA, $V_{\mathrm{mod}} = 10$ mV.)

Supplementary Sec. III. SENSITIVITY OF THE BISMUTHENE FILMS TO AND PROTECTION FROM OXIDATION

A. Oxidation effects in the photoreflectivity experiments

Monolayer bismuthene films are sensitive to oxidation and therefore have to be kept from exposure to ambient atmosphere. We have taken considerable care to protect our bismuthene films at any time by keeping the samples always under UHV-conditions or under a protective N_2 inert gas atmosphere. The first option was applied during film growth and surface analysis. Using a glovebox, the films were then transfered into a sealed metal container filled with the inert gas and equipped with a transparent window to allow for optical access.

To elucidate the importance of protection against oxidation, Supplementary Fig. S3 shows photoreflectivity (PR) spectra of bismuthene measured with the sample in inert gas atmosphere and after exposing the sample to ambient air. First, under inert gas (blue spectrum) the A-/B-resonances associated with the bismuthene excitons are observed at energies $E_\mathrm{A} \approx 1.19\,\mathrm{eV}$ and $E_\mathrm{B} \approx 1.6\,\mathrm{eV}$, respectively. This quality of the optical PR spectrum can be preserved over weeks (not shown) as long as the inert gas protection is not lifted. However, exposing the same sample to ambient air for only 10 min (orange spectrum) results in a drastic drop of the A-resonance intensity (resonance B was not measured in this experiment). After one week in ambient air (green spectrum), the PR signal is completely lost.

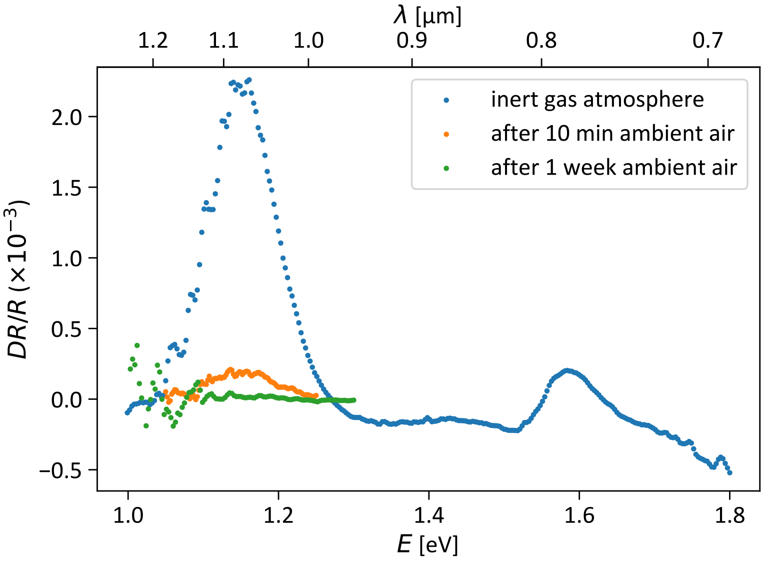

Supplementary Fig. S3. **PR spectra of bismuthene for storage under inert gas atmosphere and in ambient air conditions.** Blue data points: PR spectrum of a bismuthene sample kept in an N_2 inert gas atmosphere (< 0.1 ppm H_2O; < 0.1 ppm O_2), as for the spectrum in Fig. 2b of the main text. The spectrum shows the A-/B-resonance associated with the excitons in bismuthene centered at $E_\mathrm{A} \approx 1.19\,\mathrm{eV}$ and $E_\mathrm{B} \approx 1.6\,\mathrm{eV}$ energies, respectively. Orange data points: bismuthene PR spectrum after exposure of the sample to ambient air for 10 min. We observe a drastic decrease of the A-resonance. Green data points: PR spectrum after exposure of the sample to ambient air for 1 week. The signal of the A-resonance has completely disappeared down to the noise level.

B. XPS characterization of bismuthene oxidation and proof of efficient inert gas protection

The behavior of the PR spectra can be understood from a chemical analysis of the bismuthene film using x-ray photoelectron spectroscopy (XPS).

In Supplementary Fig. S4 we show XPS data from a bismuthene sample that has been epitaxially grown under UHV conditions, was subsequently stored in an inert gas atmosphere (< 0.1 ppm H_2O; < 0.1 ppm O_2) for 300 min, and transfered back into the UHV analysis chamber for the XPS measurements. Only a small amount of oxygen is detected on this surface, as can be seen from the intensity of the O 1s core level exceeding the background intensity by only a tiny amount in Supplementary Fig. S4b. Moreover, the core level spectrum of the spin-orbit split Bi 4f levels in

Supplementary Fig. S4c contains only a single 4f-doublet, indicative of pristine, i.e., non-oxidized Bi.

Figure S5 shows XPS spectra after exposure to ambient air for 5 min. A clear surface oxidation can be deduced from a strong O 1s signal (see overview spectrum Supplementary Fig. S5a), displaying a decomposition into two oxygen bonding states (Supplementary Fig. S5b). Even more importantly, the Bi 4f core level spectrum in Supplementary Fig. S5 c shows the dominating appearance of an additional 4f doublet, chemically shifted by ≈ 2.5 eV to higher binding energy with respect to the intrinsic Bi 4f doublet. This component is known to be a clear indicator for oxidized bismuth. We thus conclude that the short exposure to ambient air is sufficient to fully oxidize the Bi monolayer (BiO_x) and thus destroy our original bismuthene. Consequently, the optical PR data of such a sample can no longer reflect the excitonic spectra of intrinsic bismuthene. Conversely, the absence of any oxidized Bi 4f components in Supplementary Fig. S4c demonstrates that the N_2 inert gas atmosphere represents an efficient protection against unwanted bismuthene oxidation.

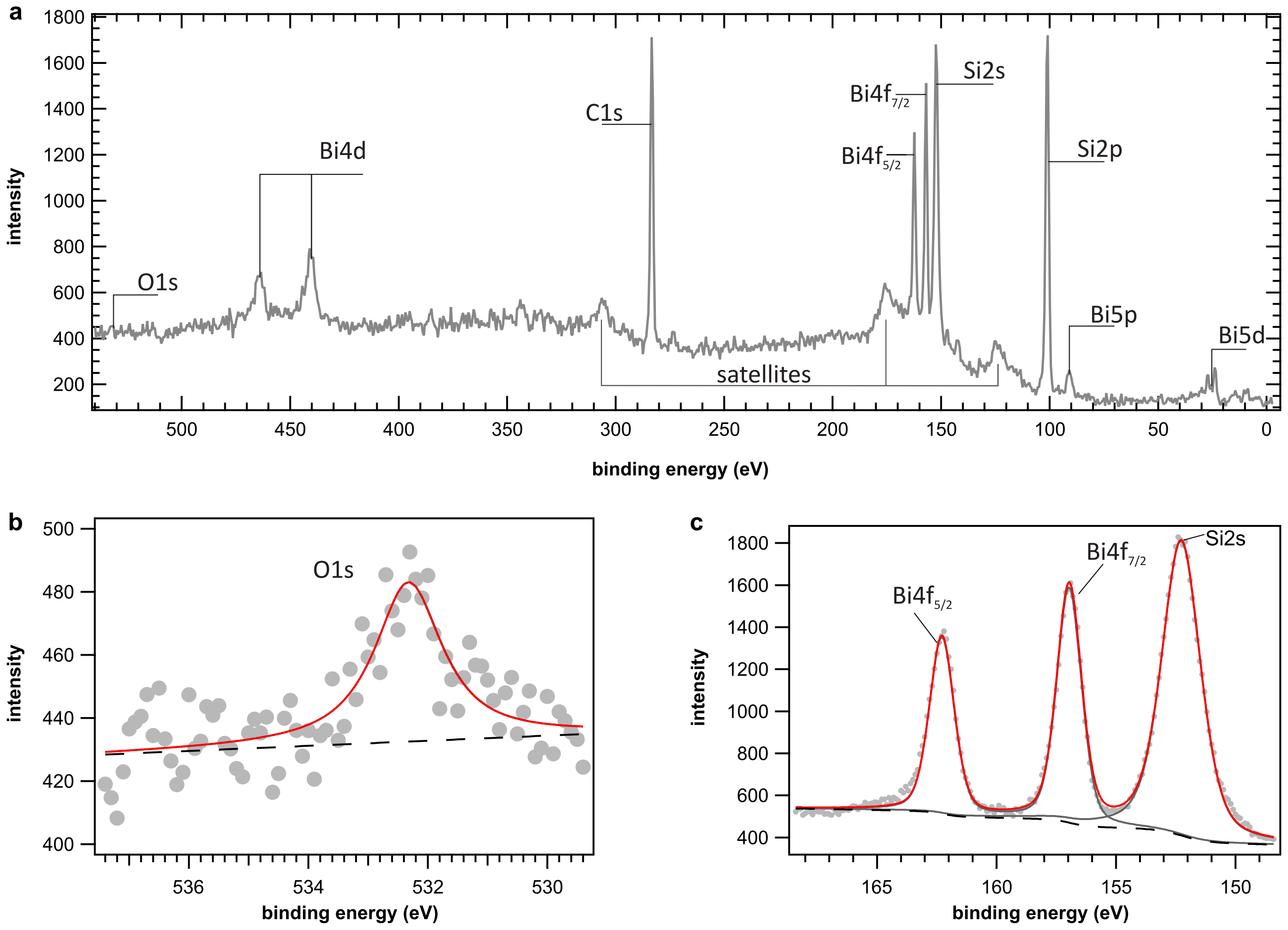

Supplementary Fig. S4. **XPS spectra of a bismuthene film after storage in inert gas atmosphere.** All XPS spectra were measured after a storage of the bismuthene sample in an N_2 inert gas atmosphere (< 0.1 ppm H_2O; < 0.1 ppm O_2) for 300 min. **a,** Overview spectrum. **b,** Gray data points: spectrum of the O 1s core level. Red line: best fit to the data using a Shirley background and a single Voigt profile. **c,** Gray data points: spectrum for the spin-orbit split Bi 4f and Si 2s core levels. Red line: best fit to the data using a Shirley background and Voigt profiles. No relevant contributions due to Bi oxidation are observed. Scan parameters: XPS spectra were measured at RT using the Al K_α line.

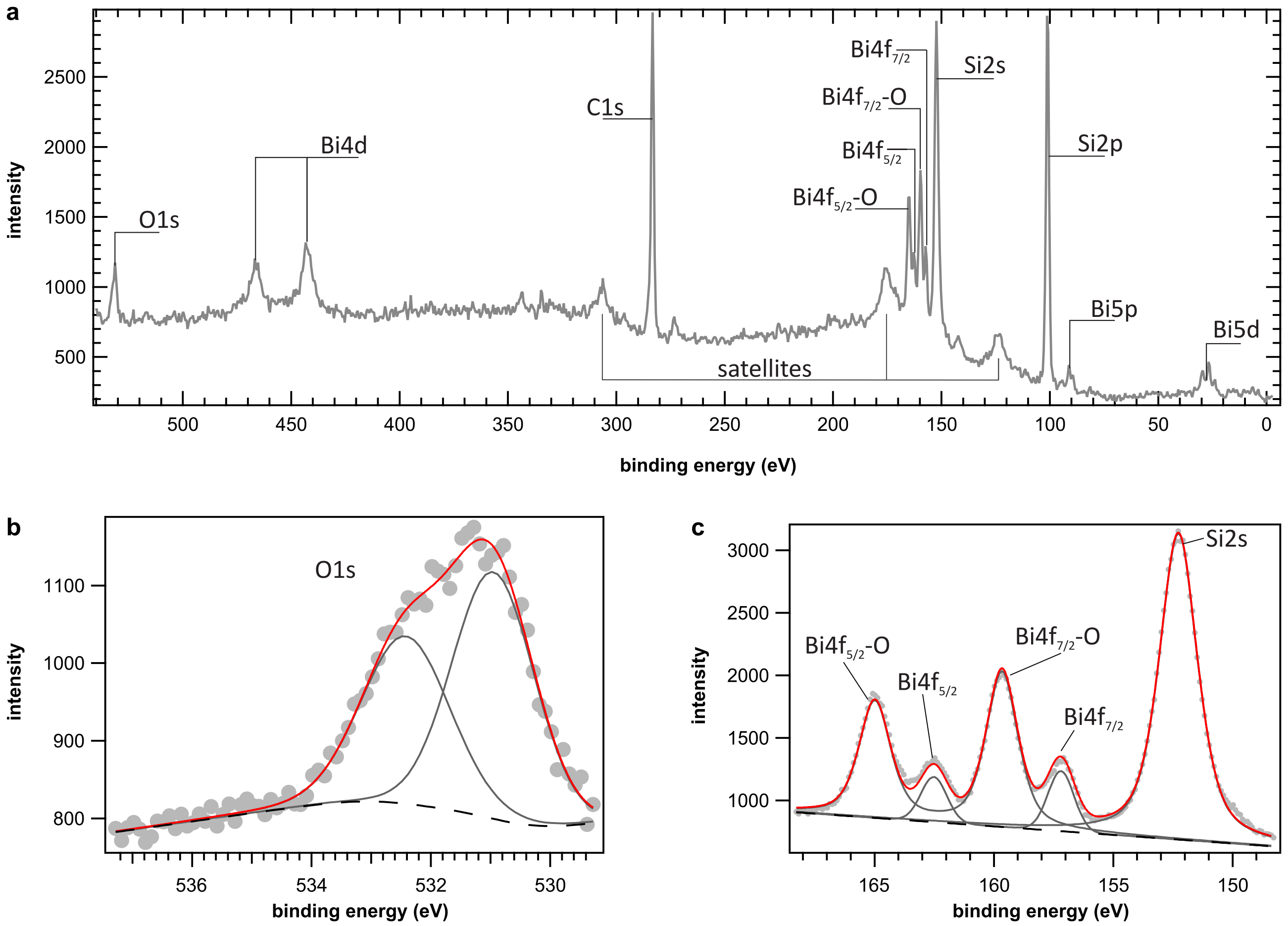

Supplementary Fig. S5. **XPS spectra of a bismuthene film after exposure to ambient air.** The bismuthene film has been exposed to ambient air for 5 min, reinserted to the UHV analysis chamber, and annealed at 250 °C for 10 min (standard surface cleaning procedure for bismuthene samples). **a,** Overview spectrum. **b,** Gray data points: spectrum of the O 1s core level. Red line: best fit to the data using a Shirley background and two Voigt profiles. The presence of a new oxygen chemical binding state compared to Fig. S4 b is observed. **c,** Gray data points: spectrum for the spin-orbit split Bi 4f and Si 2s core levels. Red line: best fit to the data using a Shirley background and Voigt profiles. Large Bi-Ox peaks are observed indicating that the bismuthene film is sensitive to oxidation. Scan parameters: XPS spectra were measured at RT using the Al K_α line.

C. Confirmation of efficient inert gas protection by valence band ARPES

Further support for the efficient inert gas protection against oxidation comes from angle-resolved photemission spectroscopy (ARPES) of the bismuthene valence band. Supplementary Fig. S6a displays an ARPES band map of an as-grown bismuthene film which never left the UHV conditions of the analysis chamber. The data is recorded in the vicinity of the K-point of bismuthene's Brillouin zone and shows the band dispersion of the Rashba-split valence band around its maximum at K. The corresponding theoretical band structure from GW calculations is depicted as green curves and shows excellent correspondence to the measured dispersion. The splitting of the valence bands from the Rashba type spin-orbit interaction amounts to $\approx 0.5\,\text{eV}$.

For comparison, we show in Supplementary Fig. S6b similar ARPES spectra of a bismuthene sample that has been stored in an N_2 inert gas atmosphere and subsequently transfered back to UHV. Prior to the ARPES measurement a standard temperature annealing process was applied, which is known to leave the bismuthene monolayer unchanged but removes physisorbed adsorbates from the surface. We note that this treatment is not capable of removing chemisorbed oxygen, i.e., once the bismuthene has been oxidized it is not possible to recover it with thermal treatment. We find that the resulting ARPES data still show the same valence band dispersion as the pristine bismuthene film. This demonstrates that even extended exposure to the N_2 inert gas atmosphere does not alter the electronic structure of our bismuthene monolayers, fully consistent with the XPS results presented above. The inert gas storage employed in our optical experiments is thus established to provide efficient protection against oxidation, ensuring that that the PR spectra recorded in this work reflect the true optical response from an intact bismuthene monolayer.

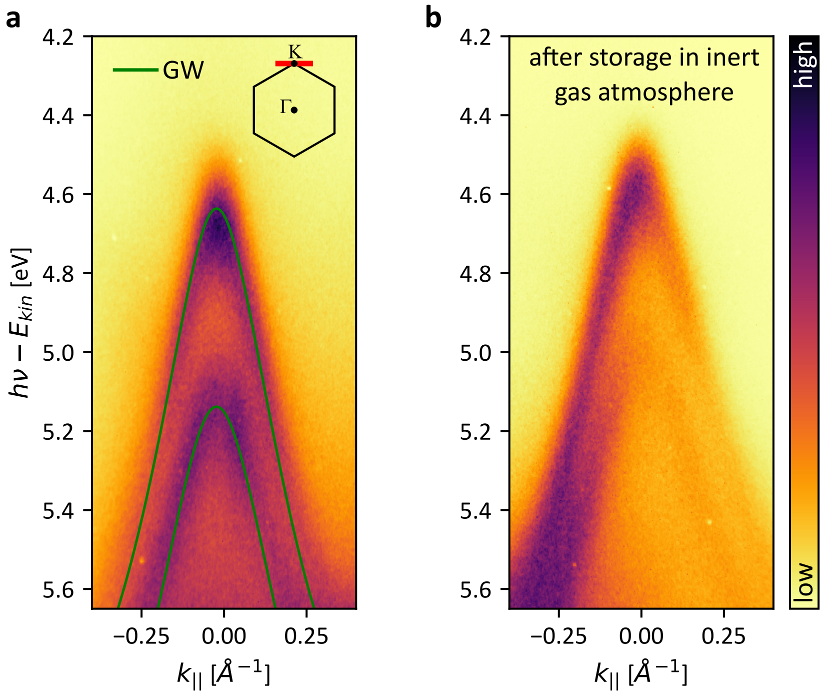

Supplementary Fig. S6. **ARPES spectra of Rashba split valence bands in bismuthene.** **a,** ARPES spectrum of the bismuthene valence band maximum at the K-point (see inset for the measured k-space path and its location relative to the Brillouin). The overlaid green curves represent the theoretical band structure from the GW calculation. (green lines). **b,** ARPES spectrum after storage of the bismuthene sample in an N_2 inert gas atmosphere (<0.1 ppm H_2O; <0.1 ppm O_2) for 90 min and reinsertion into the UHV analyis chamber followed by a standard surface cleaning procedure for bismuthene samples by annealing at 250 °C for 10 min. Scan parameters: photon energy $h\nu = 21.2\,\text{eV}$ (He Iα). Temperature in **a**: $T = 10\,\text{K}$; in **b**: T =RT.

Supplementary Sec. IV. DERIVATION OF THE PHOTOREFLECTIVITY LINESHAPE

The lineshape employed for a detailed analysis of our PR spectra is based on an adapted model originally suggested by Shanabrook *et al.* (1987) [S1]. The photo-modulated reflectivity line shape can be described phenomenologically in terms of real (ϵ_r) and imaginary (ϵ_i) parts of the complex dielectric function $\epsilon = \epsilon_r + i\epsilon_i$ as

$$\frac{\Delta R}{R} = \frac{1}{R}\frac{\mathrm{d}R}{\mathrm{d}\epsilon_r}\Delta\epsilon_r + \frac{1}{R}\frac{\mathrm{d}R}{\mathrm{d}\epsilon_i}\Delta\epsilon_i = \alpha\cdot\Delta\epsilon_r + \beta\cdot\Delta\epsilon_i, \qquad \text{(Supp. Eq. 1)}$$

where α and β are Seraphin coefficients [S2], $\Delta\epsilon_r$ and $\Delta\epsilon_i$ are ϵ_r and ϵ_i differentials. The fractional coefficients α and β are wavelength dependent, and can be derived analytically from the Fresnel reflection equation.

For the excitonic transition the complex dielectric function can be assumed to be the Lorentzian-type

$$\epsilon_r + i\epsilon_i \sim 1 + \frac{I}{E - E_g + i\Gamma}, \qquad \text{(Supp. Eq. 2)}$$

where E_g is the excitonic band-gap, Γ is transition broadening and I – intensity of the excitonic transition. In the case of the photo-modulated reflectivity experiment involving excitonic transition, the dielectric function changes $\Delta\epsilon = \Delta\epsilon_r + i\Delta\epsilon_i$ are related to the laser power P modulation and takes the first derivative functional form [S1, S3]:

$$\Delta\epsilon = \frac{\partial\epsilon}{\partial P} = \left[\frac{\partial\epsilon}{\partial E_g}\frac{\partial E_g}{\partial P} + \frac{\partial\epsilon}{\partial\Gamma}\frac{\partial\Gamma}{\partial P} + \frac{\partial\epsilon}{\partial I}\frac{\partial I}{\partial P}\right]\Delta P. \qquad \text{(Supp. Eq. 3)}$$

With $A_g = \frac{\partial E_g}{\partial P}$, $A_\Gamma = \frac{\partial\Gamma}{\partial P}$, $A_I = \frac{\partial I}{\partial P}$, and performing the partial differentiation of Supp. Eq. 2:

$$\frac{\partial\epsilon}{\partial E_g} = \frac{I}{[(E - E_g) + i\Gamma]^2}, \qquad \text{(Supp. Eq. 4)}$$

$$\frac{\partial\epsilon}{\partial\Gamma} = -\frac{iI}{[(E - E_g) + i\Gamma]^2}, \qquad \text{(Supp. Eq. 5)}$$

$$\frac{\partial\epsilon}{\partial I} = \frac{1}{(E - E_g) + i\Gamma}, \qquad \text{(Supp. Eq. 6)}$$

the dielectric function changes take the form:

$$\Delta\epsilon = \left[A_g\left(\frac{I}{[(E - E_g + i\Gamma]^2}\right) - A_\Gamma\left(\frac{iI}{[(E - E_g) + i\Gamma]^2}\right) + A_I\left(\frac{1}{(E - E_g) + i\Gamma}\right)\right]\Delta P. \qquad \text{(Supp. Eq. 7)}$$

It has been shown in Ref. [S4] and Ref. [S5] that important are only the two first terms in Supp. Eq. 7, and thus:

$$\Delta\epsilon \approx \left[A_g\left(\frac{I}{[(E - E_g) + i\Gamma]^2}\right) - A_\Gamma\left(\frac{iI}{[(E - E_g) + i\Gamma]^2}\right)\right]\Delta P. \qquad \text{(Supp. Eq. 8)}$$

By introducing the complex number $C = I\Delta P\,(A_g - iA_\Gamma) = |C|e^{i\theta}$, the Supp. Eq. 8 can be further simplified to

$$\Delta\epsilon \approx \frac{|C|e^{i\theta}}{[(E - E_g) + i\Gamma]^2}. \qquad \text{(Supp. Eq. 9)}$$

However, close to the fundamental excitonic absorption edge $\beta \approx 0$ in Supp. Eq. 1, leading to the final photo-modulated reflectivity lineshape formula for the excitonic transition:

$$\frac{\Delta R}{R} = \Re\left(|C|e^{i\theta}(E - E_g + i\Gamma)^{-2}\right). \qquad \text{(Supp. Eq. 10)}$$

Supplementary Sec. V. POWER DEPENDENCE OF THE PHOTOREFLECTIVITY MEASUREMENTS

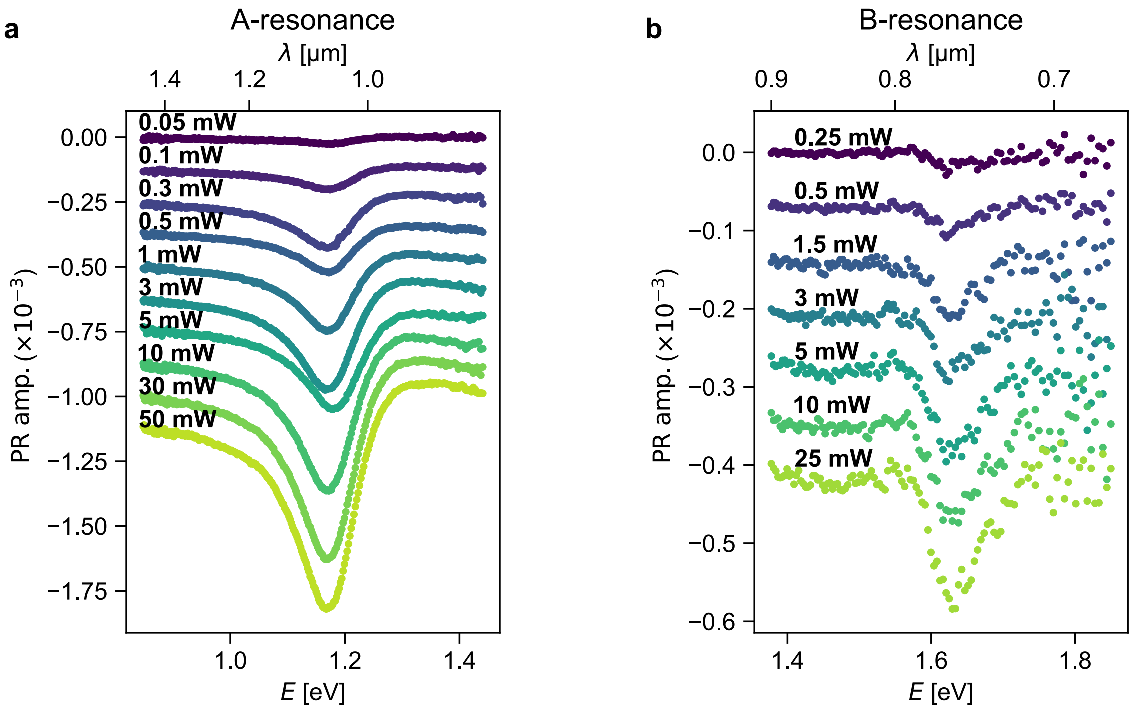

Supplementary Fig. S7. **Power dependent PR measurements of A-/B-exciton. a**, evolution of the A resonance with the averaged pumping power provided by the chopped continuous-wave neodymium-doped yttrium aluminum garnet laser emitting at 532 nm. **b**, evolution of the B resonance with the averaged pumping power from the synchronously pulse pumped optical parametric oscillator emitting at 650 nm.

Supplementary Figure S7 presents the evolution of the PR amplitude for A and B resonances with the time averaged modulation laser power (indicated). The spectra are gathered from the 0.0 mm horizontal spatial position on the bismuthene sample. For the A resonance, we use a continuous-wave (CW) neodymium-doped yttrium aluminum garnet laser emitting at 532 nm. For the B resonance the modulation is provided by an optical parametric oscillator with an emission wavelength of 650 nm. As expected, for both the A- and B- exciton signal, the strength of the PR signal monotonously increases with the modulation power which is varied over a few decades.

Supplementary Sec. VI. DETERMINATION OF SINGLE-PARTICLE BAND ONSETS FROM STS

In Fig. 1b of the main text we present a typical bismuthene $\mathrm{d}I/\mathrm{d}V$ tunneling spectrum. The observed electronic structure is characterized by a large electronic band gap and additional features in the spectrum which we labeled $V_{1,2}$ in the occupied states and $C_{1,2}$ in the unoccupied states. Here we explain the determination scheme for the values of $V_{1,2}$ and C_1.

Supplementary Figure S8a is a detailed plot of the $\mathrm{d}I/\mathrm{d}V$ of Fig. 1b around V_2. It is clearly seen that V_2 marks a kink in the spectrum which can be attributed to the onset of the lower Rashba band in the bismuthene valence band. For the numerical extrapolation of V_2 we fitted two linear functions to the data within the regions I and II (red dashed lines indicate best fits). We take the intersection point of the fitting lines as the value of V_2 and obtain $V_2 = -0.57\,\mathrm{eV}$.

Supplementary Figure S8b is a detailed log-plot of the $\mathrm{d}I/\mathrm{d}V$ of Fig. 1b around V_1. For the numerical extraction of V_1 we fitted the noise level within the single-particle band gap of bismuthene with a constant, i.e., we determined the mean tunneling intensity within the single particle gap, and the valence band edge in the yellow shaded region with a linear function (red dashed lines indicate best fits). We take the intersection point of the fitting lines as the value for V_1 and obtain $V_1 = -0.04\,\mathrm{eV}$. For the determination of the conduction band onset in Supplementary Fig. S8c we applied the same procedure as for the valence band onset in Supplementary Fig. S8b, and obtain $C_1^{\mathrm{upper}} = 0.92\,\mathrm{eV}$ as

an upper limit. For a lower limit of the conduction band onset C_1^{lower} we take the low energetic end of a hump in the $\mathrm{d}I/\mathrm{d}V$, energetically just below the steep onset of the conduction band, that is frequently observed in bismuthene tunneling spectra: we obtain $C_1^{\text{lower}} = (0.80 \pm 0.05)\,\text{eV}$.

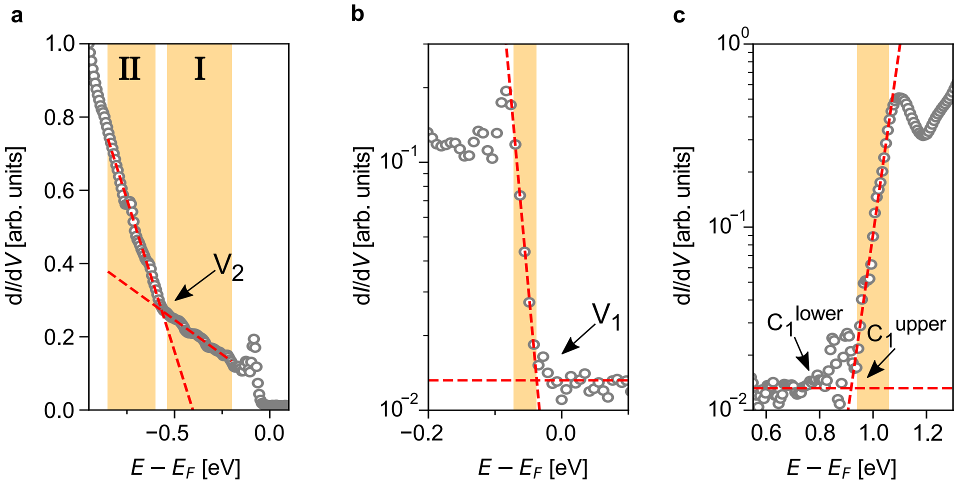

Supplementary Fig. S8. **Determination scheme for the values of $V_{1,2}$ and C_1 from STS. a,** Detailed plot of the $\mathrm{d}I/\mathrm{d}V$ of Fig. 1b around V_2 which is attributed to the onset of the lower Rashba band. Red dashed lines mark best fits to the spectrum for the determination of V_2 (see text for details). **b, c,** Detailed log-plots of the $\mathrm{d}I/\mathrm{d}V$ of Fig. 1b around V_1 and C_1, respectively. Red dashed lines mark best fits to the spectrum for the determination of V_1 and C_1 (see text for details). Scan parameters as in Fig. 1b: $T = 4.2\,\text{K}$, $V_{\text{set}} = -0.4\,\text{V}$, $I_{\text{set}} = 200\,\text{pA}$, $V_{\text{mod}} = 10\,\text{mV}$.

Supplementary Sec. VII. TOPOLOGICAL NATURE OF EXCITONS IN BI/SIC

In this section we discuss the *global* topological properties of excitons and afterward we outline the procedure for achieving a *local* topology via external fields.

i) The GLOBAL topological properties, are embedded in the in-gap poles of the linear response function, i.e. the Bethe-Salpeter equation (BSE). From this, one can compute the exciton wave function for exciton i, for all momenta $\mathbf{q}$ in the Brillouin Zone (BZ). This allows the calculation of the exciton Chern number $C(i)$ via the integral over the Berry curvature, and consequently determines whether the exciton displays a global topology or not.
In other words, the mechanism for topological excitons is similar to the band inversion in the single-particle band structure case: a topological exciton is composed of electron-hole excitations, which have different parities under inversion in different regions of the BZ. When one connects this with the Berry curvature (see e.g. Eq. 34 in [S6]), it can be seen immediately that the curvature is even under inversion and odd under time-reversal symmetry. Thus, if a system is invariant under both, the exciton Chern number vanishes by symmetry. In the case of [S6] and its inversion symmetric BHZ model for the QSH insulator, also all excitons are invariant under inversion and can be classified by $S(z)$ quantum numbers. Whereas the $S(z) = 0$ excitons are additionally invariant under time-reversal, the $S(z) = +1$ and $S(z) = -1$ map onto each other, and can have a nonzero Chern number, i.e., global topology.
In this case, the $S(z) = \pm 1$ exciton dispersion along high-symmetry paths displays even and odd parities in such a way that the dispersing excitons acquire a finite Chern number.
This procedure of verifying the exciton topology, via realizing that all excitons are invariant under inversion, and thus checking for which excitons are not simultaneously invariant under time reversal, has to be modified in our Bi/SiC system.
In the Bi/SiC system, that is, in case of the presence of the Rashba term and the inversion symmetry perpendicular to the substrate being broken, the simple symmetry argument for the BHZ model and its topological excitons does not hold anymore. Thus, we have to indeed calculate the exciton Chern number via the integral over the Berry curvature.
On the theoretical side this can in principle be achieved via available exciton computer packages, starting from the BSE of our present study of excitons in Bi/SiC.
On the experimental side, a first possibility consists in probing exciton dispersion by momentum resolved electron energy loss spectroscopy ($\mathbf{q}$-dependent EELS) [S7].
The second one is time-resolved angle-resolved photoemission, i.e. tr-ARPES.

Here the finite-momentum excitons, that lie outside the radiative light-cone, become accessible by time-and angle-resolved photoemission spectroscopy; the spectroscopic tool can indeed access to excited states including excitons, in energy-momentum space and on ultra fast time scales [S8]. Both experiments are rather challenging and thus time-consuming.

ii) The LOCAL topological properties are due to the fact that the conventional optical selection rules, determining the exciton envelope functions at a k-point, are not valid for 2D systems with nontrivial band topology: they have to be replaced by a formula incorporating topological effects [S9, S10].
As a consequence, in transition-metal dichalcogenides (TMDCs) and graphene-based systems, a mapping exists between band topology around the K and K' valleys and optical selection rules, which can be described in terms of a so-called gapped chiral fermion model. In these chiral-fermion systems [S9], the winding number characterizing the topology of the Bloch bands at K and K' determines the allowed optical transitions and the excitonic properties. Thus, anomalous exciton selection results have been found, such that both the s-like and d-like excitons are bright in monolayer MoS_2, with their optical transitions having opposite circular polarization (σ^+, σ^-). The crucial point is that, in TMDCs, one has in each valley (K or K') two decoupled chiral fermion systems with the same winding numbers. This is why TMDCs exhibit valley selective optical selection rules.
In Bismuthene, on the other hand, it can be shown [S11] that each valley supports chiral fermion models with different winding numbers.
Hence, there are exciton/excitations coupling to σ^+ and σ^- in each valley.
What can be done, following the procedure outlined in [S11], is a reordering of the excitons via external fields (magnetic (possibly via magnetic dopants) field plus electric field) in our Bismuthene system, where one focuses only on the lowest exciton series.
In the end, this induces a change of the winding number from $w = 1$ to $w = -1$, say in the K' valley, which transforms the series of bright excitons with respect to the helicity of light.
As an example: the 1s exciton with angular quantum number $m = 0$ is bright under σ^+ when the winding number $w = 1$. If, on the other hand, $w = -1$, it is, instead bright under σ^-.
In summary, one can, at least in principle, optically probe local topology in Bismuthene via gate-controlled band ordering through changes in the valley winding numbers. This can experimentally be detected in helicity resolved absorption and photoluminescence.

[S1] Shanabrook, B. V., Glembocki, O. J. & Beard, W. T. Photoreflectance modulation mechanisms in GaAs-$Al_xGa_{1-x}As$ multiple quantum wells. *Phys. Rev. B* **35**, 2540 (1987). URL https://link.aps.org/doi/10.1103/PhysRevB.35.2540.

[S2] Seraphin, B. O. & Bottka, N. Band-Structure Analysis from Electro-Reflectance Studies. *Phys. Rev.* **145**, 628 (1966). URL https://link.aps.org/doi/10.1103/PhysRev.145.628.

[S3] Enderlein, R., Jiang, D. & Tang, Y. On the mechanisms of photoreflectance in multiple quantum wells. *Phys. Stat. Sol.(b)* **145**, 167 (1988). URL https://onlinelibrary.wiley.com/doi/abs/10.1002/pssb.2221450114.

[S4] Pollak, F. H. & Glembocki, O. J. Modulation Spectroscopy Of Semiconductor Microstructures: An Overview. In Glembocki, O. J., Pollak, F. H. & Ponce, F. A. (eds.) *Spectroscopic Characterization Techniques for Semiconductor Technology III*, vol. 0946, 2 – 35. International Society for Optics and Photonics (SPIE, 1988). URL https://doi.org/10.1117/12.947409.

[S5] Glembocki, O. & Shanabrook, B. Temperature dependence of photoreflectance line shapes in GaAsAlGaAs multiple quantum wells. *Superlattices and Microstructures* **3**, 235–238 (1987). URL https://www.sciencedirect.com/science/article/pii/0749603687900644.

[S6] Blason, A. & Fabrizio, M. Exciton topology and condensation in a model quantum spin hall insulator. *Phys. Rev. B* **102**, 035146 (2020). URL https://link.aps.org/doi/10.1103/PhysRevB.102.035146.

[S7] Cudazzo, P., Sottile, F., Rubio, A. & Gatti, M. Exciton dispersion in molecular solids. *J. Phys.: Condens. Matter* **27**, 113204 (2015). URL https://doi.org/10.1088/0953-8984/27/11/113204.

[S8] Dong, S. *et al.* Direct measurement of key exciton properties: Energy, dynamics, and spatial distribution of the wave function. *Natural Sciences* **1**, e10010 (2021). URL https://onlinelibrary.wiley.com/doi/abs/10.1002/ntls.10010.

[S9] Zhang, X., Shan, W.-Y. & Xiao, D. Optical Selection Rule of Excitons in Gapped Chiral Fermion Systems. *Phys. Rev. Lett.* **120**, 077401 (2018). URL https://link.aps.org/doi/10.1103/PhysRevLett.120.077401.

[S10] Cao, T., Wu, M. & Louie, S. G. Unifying Optical Selection Rules for Excitons in Two Dimensions: Band Topology and Winding Numbers. *Phys. Rev. Lett.* **120**, 087402 (2018). URL https://link.aps.org/doi/10.1103/PhysRevLett.120.087402.

[S11] Xu, G., Zhou, T., Scharf, B. & Žutić, I. Optically Probing Tunable Band Topology in Atomic Monolayers. *Phys. Rev. Lett.* **125**, 157402 (2020). URL https://link.aps.org/doi/10.1103/PhysRevLett.125.157402.
